# Supplementary material for: An ensemble machine learning model based on multiple filtering and supervised attribute clustering algorithm for classifying cancer samples
Source: PeerJ Comput Sci. 2021 Sep 16;7:e671. doi: 10.7717/peerj-cs.671 (PMC8459790; doi:10.7717/peerj-cs.671)
Supplement: Supplemental Information 1 — Each row of this table illustrates one of the seven filter methods used in the proposed MFSAC-EC model. Every equation along with the description defines every filer score function. [file peerj-cs-07-671-s001.docx]

**Table S1. Description of Different Filter Methods**

| **Filter Method** | **Definition** | **Description** |
| --- | --- | --- |
| Modified Fisher score (Gu 2011) | $F\left( G_{t} \right)= \frac{\sum_{j=1}^{N} n_{j}\left( m_{t}^{j}-m_{t} \right)^{2}}{\left( \sigma_{t} \right)^{2}} (1)$ | Here modified Fisher score of feature $G_{t}$ is presented.  Here $n_{j}$ represents the number of samples of class $j$, $m_{t}^{j}$ and $\sigma_{t}^{j}$are the mean and standard deviation of $t$ th feature for $j$ th class respectively, while $m_{t}$ and $\sigma_{t}$ are the mean and standard deviation of $t$ th feature$, where \left( \sigma_{t} \right)^{2}= \sum_{j=1}^{N} \left( \sigma_{t}^{j} \right)^{2}$ . |
| Modified T-test (Zhou 2007) | t($G_{t})=\max\{\frac{m_{t}^{j}-m_{t}}{M_{j}S_{t}}, j=1,\ldots., N$ (2) | Here modified t-test of feature $G_{t}$ is presented.  Here, $m_{t}^{j}$ and $m_{t}$ are the mean of $t$th feature for $j$th class and mean of $t$th feature respectively. $S_{t}$ is the sum of within class standard deviation and is represented as,  $S_{t}^{2}= \frac{1}{U-N}\sum_{j=1}^{N} \sum_{l\in j} \left( x_{lt}-m_{t}^{j} \right)^{2},$  While  $M_{j}=sqrt( \frac{1}{n_{j}}+ \frac{1}{U})$, $n_{j}$ is the number of samples for $j$th class, $x_{lt}$is lth sample’s value for $t$ th feature in $j$th class. Here $U$and $N$ are total number of samples and total number of classes respectively. |
| Chi Square (Das 2019) | $\chi^{2}\left( G_{t}, C \right)=\sum_{l\in L} \sum_{k=1}^{N} \frac{\left( (observed\left( l,d_{j} \right)-expected(l,d_{j}) \right)^{2}}{expected(l,d_{j})} (3)$ | Chi Square is a statistical test which is commonly used to compare observed data with the expected data according to a specific hypothesis. This test is also used as a measure to find class discrimination capability of a gene with respect to class vector. The chi square value of every gene vector is calculated in equation (3).  Here, $L$ is the set of all distinct values present in a gene, if the gene is discretized. $observed\left( l,d_{j} \right)$ and $expected(l,d_{j})$ represent the number of observed and expected co-occurrence of value $l$ and $d_{j}$ appeared in gene $G_{t}$ and class vector $C$ respectively. |
| Mutual Information (Das 2019) | $MInfo\left( G_{t}, C \right)= \sum_{d_{k}\in DC} \sum_{l\in L} P(l, d_{j})log\frac{P(l, d_{j})}{(P\left( l \right)P\left( d_{j} \right))} (4)$  Mutual information is also represented using entropy measure shown in equation (5).  $MInfo\left( G_{t}, C \right)=H\left( G_{t} \right)-H\left( G_{t} \vert C \right) (5)$ | Mutual information is an important measure based on information theory. Mutual information between a gene and a class vector can be defined as in equation (4).  Here,  $H\left( G_{t} \right)$ is the entropy or amount of information present in$G_{t}$ and $H\left( G_{t}\vert C \right)$ is the entropy or amount of information present in $G_{t}$ in presence of $C$. Here, $L$ is the set of all distinct values present in a gene vector, if the gene vector is discretized. |
| Relief-F (Das 2019) | $RE\left( G_{t} \right)=RE\left( G_{t} \right)-\sum_{m=1}^{K} \frac{diff\left( G_{t}, R_{i}, H_{m} \right)}{w.K}+\sum_{c_{j}\neq class\left( R_{i} \right)} \frac{\left[ \frac{P\left( c_{j} \right)}{1-P\left( class\left( R_{i} \right) \right)} \sum_{m=1}^{K} diff\left( G_{t}, R_{i}, M_{m} \right) \right]}{w.K} (6)$ | Relief-F measures class discrimination power of a feature according to its distinguishing capability between near instances. For any random instance $R_{i}$, ReliefF finds the nearest instance from same class to find a hit $(H)$ and a miss $(M)$ from different class and according to that $RE\left( G_{t} \right)$ is increased or decreased using equation (6). This process is run for w number of times for different instances. |
| Pearson Correlation Coefficient (Leung 2010) | $\rho(x, y)= \frac{\sum_{i} (x_{i}-\bar{x})(y_{i}-\bar{y})}{\surd\sum_{i} {(x_{i}-\bar{x})}^{2}{(y_{i}-\bar{y})}^{2}}$ (7) | Pearson Correlation is used for detecting the linear relationship between two vectors. The equation (7) is used to calculate the PC (ρ) between the independent vector x and dependent vector y. For this paper x is considered as $G_{t}$ and y as class vector C. |
| Signal-to-noise ratio (Leung 2010) | $S/R\left( G_{t} \right)= \frac{\sum_{j=1}^{N} (m_{j}^{t}-m_{t})}{\sigma_{t}} (8)$ | The signal to noise ratio test is a feature selection method that selects significant features according to their expression levels using S/R test.  Here $m_{t}^{j}$ and $\sigma_{t}^{j}$are the mean and standard deviation of $t$ th feature for $j$ th class respectively, while $m_{t}$ and $\sigma_{t}$ are the mean and standard deviation of $t$ th feature$, where \sigma_{t} = \sum_{j=1}^{N} \sigma_{t}^{j}$ |
