# Supplementary figures and images for: An ensemble machine learning model based on multiple filtering and supervised attribute clustering algorithm for classifying cancer samples
Source: PeerJ Comput Sci. 2021 Sep 16;7:e671. doi: 10.7717/peerj-cs.671 (PMC8459790; doi:10.7717/peerj-cs.671)

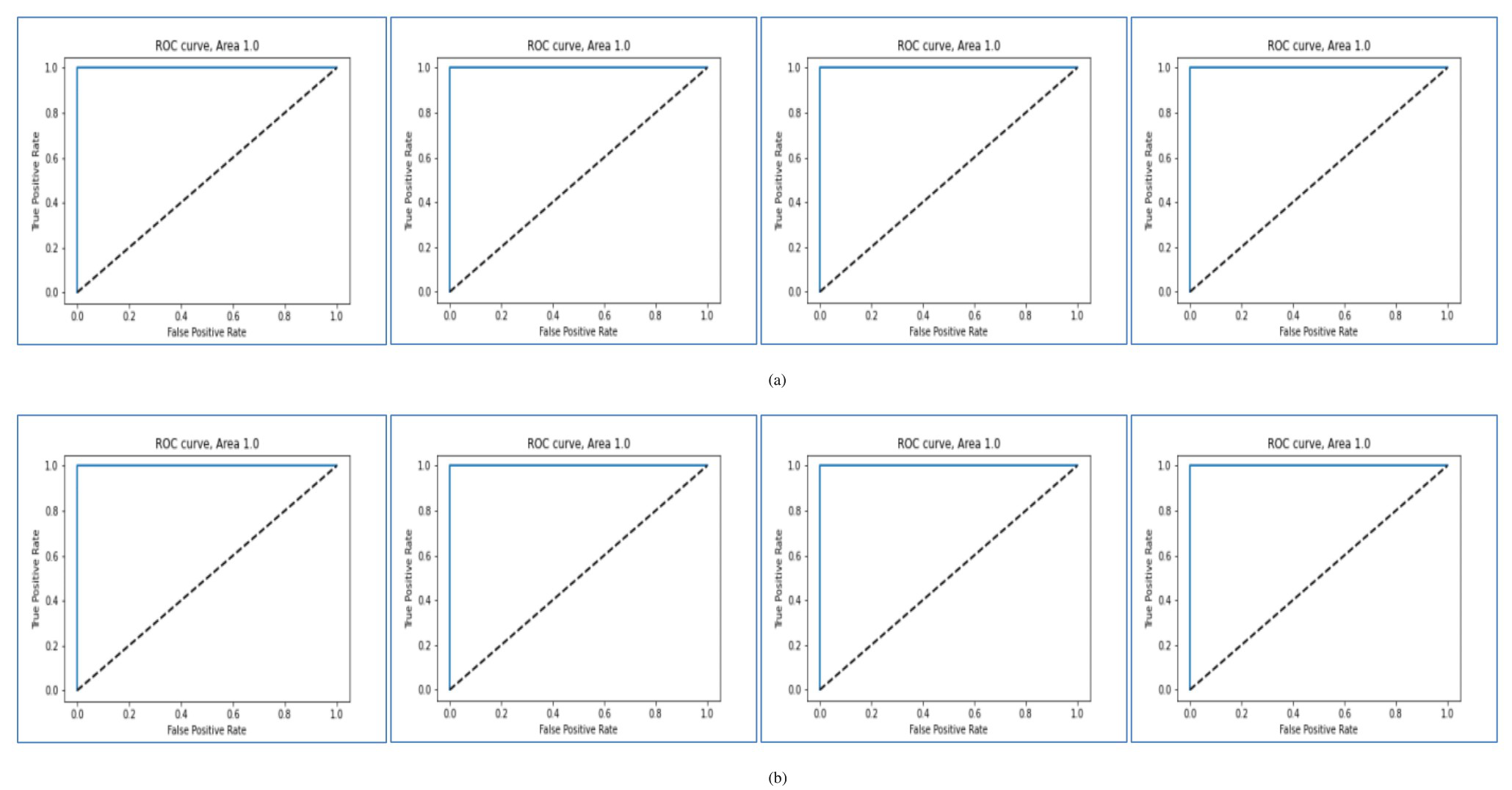

Supplement: Supplemental Information 2 — For (a) Leukemia Cancer (b) Lung Cancer datasets. [file peerj-cs-07-671-s002.png]

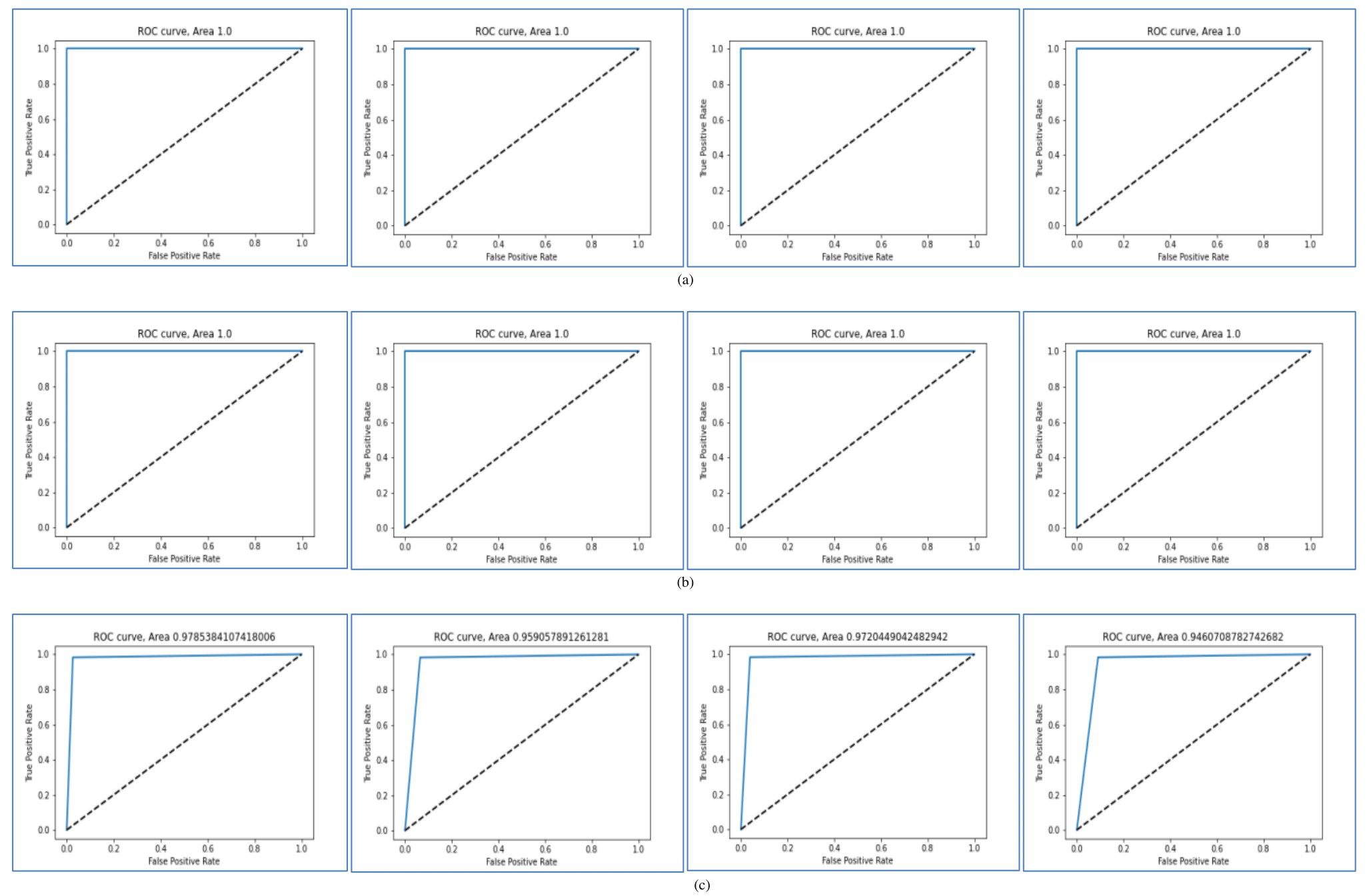

Supplement: Supplemental Information 3 — For (a)RAOA dataset using fivefold. (b) RBreast dataset using fivefold. (c) Prostate Cancer dataset using tenfold. [file peerj-cs-07-671-s003.png]

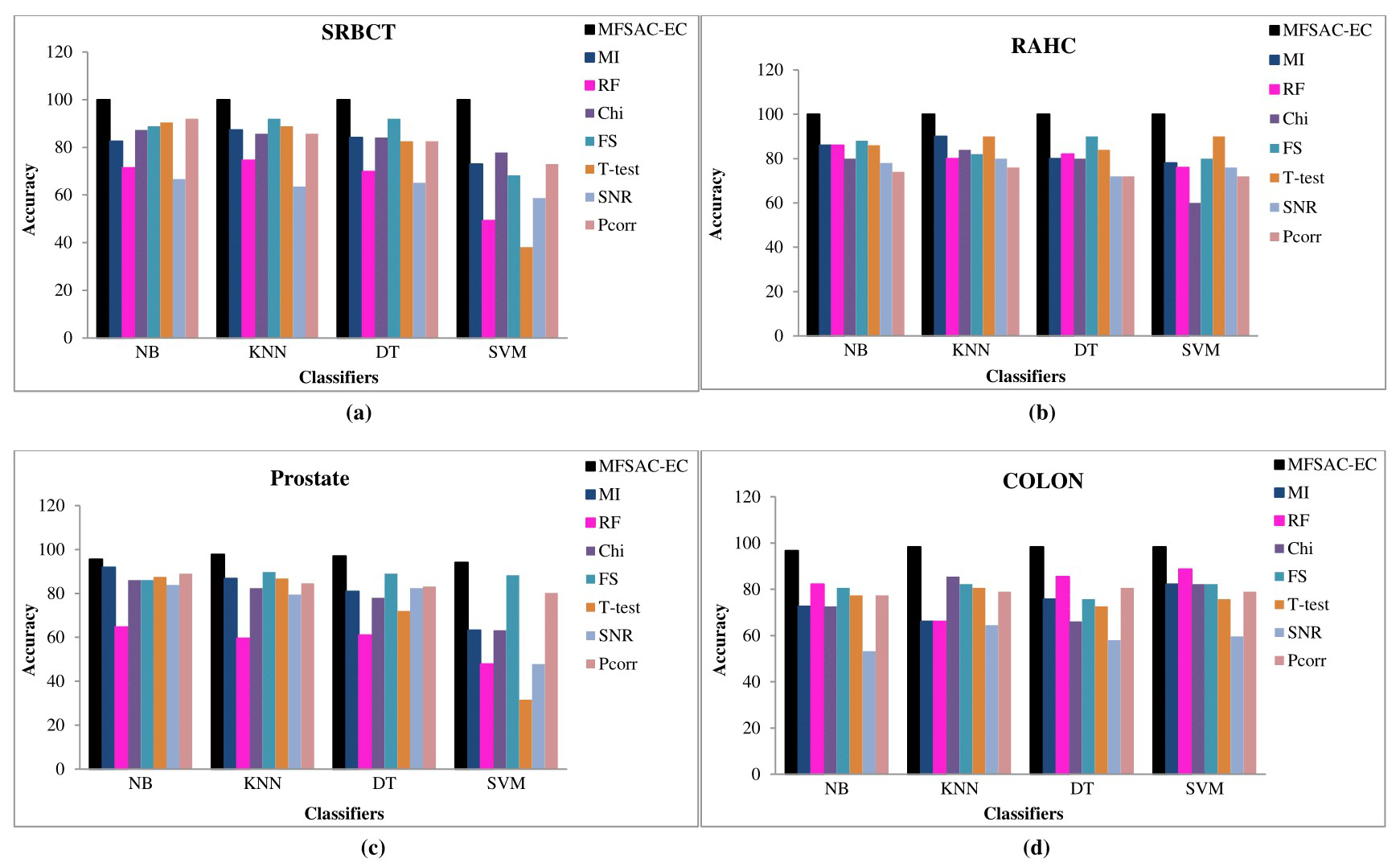

Supplement: Supplemental Information 4 — Classification accuracy(%) of MFSAC-EC model along with seven filter methods using classifiers Naive Bays, KNN, Decision Tree (C4.5), and SVM are represented using different colored bars respectively for (a) SRBCT (b) RAHC (c) Prostate and (d) Colon Cancer datasets. [file peerj-cs-07-671-s004.png]

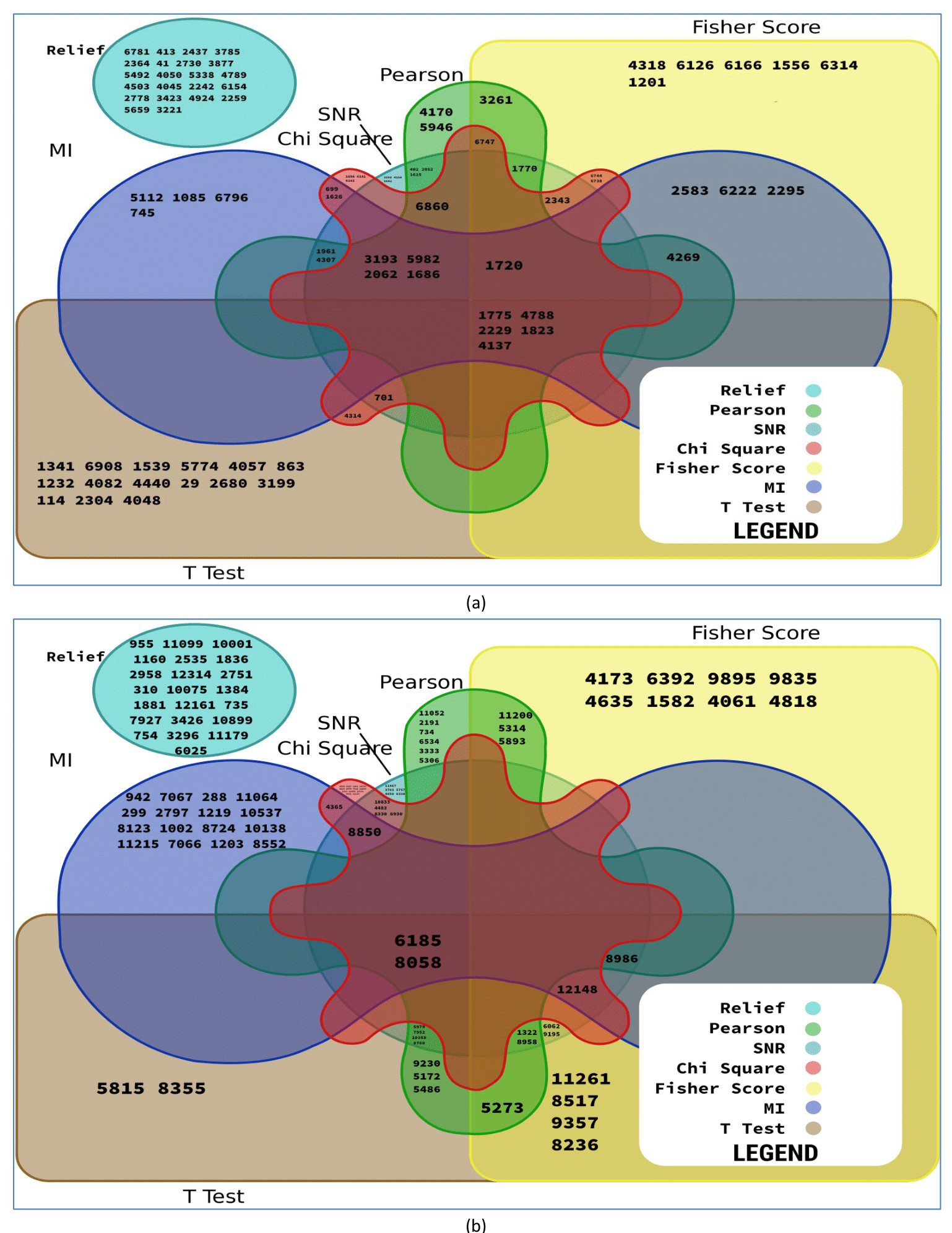

Supplement: Supplemental Information 5 — (a) Leukemia Cancer dataset. (b) Prostate Cancer dataset. [file peerj-cs-07-671-s005.png]
